# Measuring Structural Racism to Advance Health Equity in Aging

**DOI:** 10.1093/geroni/igaf122.1473

**Published:** 2025-12-31

**Authors:** Karen Bandeen-Roche, Boeun Kim, Roland Thorpe

## Abstract

Structural racism is a fundamental determinant of health inequities among older adults, yet its complexity poses significant challenges for research. This symposium brings together a panel of interdisciplinary researchers with expertise in biostatistics, nursing, epidemiology, sociology, and public health to explore innovative approaches to measuring structural racism and understanding its impact on aging. Dr. Paris Adkins-Jackson of Columbia University will discuss an autoethnographic study reflecting on a multidisciplinary team’s approach to measuring structural racism, highlighting challenges and opportunities in this research. Dr. Karen Bandeen-Roche of Johns Hopkins University will present a novel modeling framework for measuring structural racism that analyzes racial disparities in healthcare, education, and policing to provide a better understanding of systemic inequities. Dr. Boeun Kim of University of Iowa will examine the impact of residential segregation and economic deprivation on mental health among older adults, highlighting how racialized economic segregation differentially affects Black and White individuals across the life course. Dr. Erik Westlund of Johns Hopkins University will highlight challenges in processing and sharing publicly available data to study structural racism, addressing issues such as recency bias and data integration. Dr. Roland J. Thorpe Jr. of Johns Hopkins University will serve as the discussant, leading a discussion on bridging research, policy, and practice to advance structural racism research in aging. This session will provide critical insights into methodological advancements, interdisciplinary collaboration, and the translation of research into action to drive meaningful change in aging and health equity.

